# Monosynaptic functional connectivity in cerebral cortex during wakefulness and under graded levels of anesthesia

**DOI:** 10.3389/fnint.2012.00090

**Published:** 2012-10-12

**Authors:** Jeannette A. Vizuete, Siveshigan Pillay, Kamran Diba, Kristina M. Ropella, Anthony G. Hudetz

**Affiliations:** ^1^Department of Biomedical Engineering, Marquette UniversityMilwaukee, WI, USA; ^2^Department of Biophysics, Medical College of WisconsinMilwaukee, WI, USA; ^3^Department of Psychology, University of Wisconsin at MilwaukeeMilwaukee, WI, USA; ^4^Department of Anesthesiology, Medical College of WisconsinMilwaukee, WI, USA

**Keywords:** consciousness, cross-correlogram analysis, cortical monosynaptic connectivity, excitatory–inhibitory balance, connection strength

## Abstract

The balance between excitation and inhibition is considered to be of significant importance for neural computation and cognitive function. Excitatory and inhibitory functional connectivity in intact cortical neuronal networks in wakefulness and graded levels of anesthesia has not been systematically investigated. We compared monosynaptic excitatory and inhibitory spike transmission probabilities using pairwise cross-correlogram (CCG) analysis. Spikes were measured at 64 sites in the visual cortex of rats with chronically implanted microelectrode arrays during wakefulness and three levels of anesthesia produced by desflurane. Anesthesia decreased the number of active units, the number of functional connections, and the strength of excitatory connections. Connection probability (number of connections per number of active unit pairs) was unaffected until the deepest anesthesia level, at which a significant increase in the excitatory to inhibitory ratio of connection probabilities was observed. The results suggest that the excitatory–inhibitory balance is altered at an anesthetic depth associated with unconsciousness.

## Introduction

Local computations within neuronal networks constitute the foundation for information processing that ultimately leads to conscious experience and behavior (Buzsaki, 2006, 2007; Buzsaki et al., 2007). The balance between excitation and inhibition in local networks is also considered to be of significant importance for neural computation, cognitive function, and regulation of global firing activity (Bartho et al., 2004; Buzsaki, 2006, 2007). Parallel recording of extracellular activity using microelectrodes is the principal technique to investigate neuronal communication within localized areas. Accordingly, there has been a strong interest in reliable approaches to extract neuronal connectivity from multichannel unit recordings in both awake and anesthetized animals. How the derived neuronal interactions depend on the level of consciousness including waking, sleep, and anesthesia is a principal question that may shed light on the neuronal mechanisms underlying neuronal computations that support cognitive functions. To date, relatively little is known about the nature of anesthetic dose-dependent changes in functional interactions in intact neuronal networks. The modulation of neuronal communication by anesthetic agents is of particular interest because anesthetics can be applied to investigate the emergence and breakdown of consciousness in a controlled manner.

Several studies suggest that the brain's ability to process and integrate information across remote and local areas in the cerebral cortex gives rise to conscious experience (Tononi and Edelman, 1998; Alkire et al., 2008). We suggested that long-range functional communication within the cerebral cortex is disrupted during loss of consciousness as produced by various anesthetics (Hudetz, 2002; Hudetz et al., 2003; Imas et al., 2005a,b, 2006). Likewise, a loss of cortical effective connectivity has been demonstrated in humans at an anesthetic depth associated with unconsciousness (Lee et al., 2009; Ferrarelli et al., 2010; Langheim et al., 2011). Furthermore, a recent study using local field potential recordings, found a concentration-dependent effect of several anesthetics on intracortical functional connections, suggesting that anesthetics modulate neuronal communication in local circuits (Kreuzer et al., 2010). Thus, functional communication in neuronal networks may be a primary target of anesthetics.

Anesthetic agents have been shown to exert graded suppressive effects on both spontaneous and evoked neuronal activity (Detsch et al., 2002; Villeneuve and Casanova, 2003; Hudetz et al., 2009; Sleigh et al., 2009). Moreover, most common anesthetics suppress excitatory and facilitate inhibitory synaptic transmission (Pearce et al., 1989; Pittson et al., 2004). Whereas the effect of anesthesia on single unit activity (UA) has been studied extensively, how the observed synaptic changes influence communication in the intact neuronal network remains unclear. Elucidation of the latter requires an estimation of functional neuronal connectivity from the simultaneous recording of a large number of active units, *in vivo*, across multiple states of arousal.

Numerous techniques have been recently applied to estimate functional connectivity in intact neuronal networks (Brown et al., 2004; Kass et al., 2005). Putative monosynaptic connections can be identified in local networks of extracellularly recorded units by estimating spike transmission probabilities from cross-correlogram (CCG) analyses (Csicsvari et al., 1998; Bartho et al., 2004; Fujisawa et al., 2008). Results showed that spike transmission probabilities were state-dependent in rat hippocampal cells: highest during exploration and rapid-eye movement (REM) sleep, as observed by the presence of theta waves, and lowest during sharp-wave bursts associated with slow-wave sleep (Csicsvari et al., 1998). Fujisawa et al. further demonstrated behavior-dependent changes in short-term functional connectivity as measured by monosynaptic interactions in the medial prefrontal cortex (Fujisawa et al., 2008). These studies demonstrate that the efficacy of spike transmission within a neural network may depend on brain state, and consequently, the animal's level of consciousness.

The studies conducted using CCG analysis have been mainly performed in intact cortical neuronal networks during wakefulness, sleep or deep anesthetic levels (McGaraughty and Reinis, 1993; Csicsvari et al., 1998; Bartho et al., 2004; Fujisawa et al., 2008; Fujiwara et al., 2008). However, deep anesthesia associated with nociceptive immobility (Rampil, 1994; Antognini and Kien, 1995) does not inform us about dose-dependent changes associated with the loss and return of consciousness (Gugino et al., 2001). To understand the critical changes in network function associated with loss of consciousness, there is a need to determine, in a controlled manner, how spike transmission probabilities are altered at multiple graded levels of anesthesia. In this study, we compare excitatory and inhibitory spike transmission probabilities in rat cerebral cortex during wakefulness and under graded levels of anesthesia.

## Results

### Behavioral observations

Experiments were performed on seven rats at three levels of inhaled desflurane anesthesia (6, 4, and 2%) and wakefulness. At the 6% level, spontaneous movement was absent. As the anesthetic was withdrawn, rats exhibited a gradual increase in their level of alertness. At moderate depth of anesthesia (4%), they displayed sporadic and brief behaviors such as, temporary whisker twitching or chewing, but for the most part, they remained immobile. During light sedation (2%), most rats displayed head and limb movements, and postural changes that lasted for several seconds. Finally, during wakefulness (0%), rats displayed typical intermittent grooming and exploratory behaviors as well as quiet (absence of movement) alertness. The return of righting reflex suggested that consciousness was regained at 4% anesthetic concentration.

### Unit activity and monosynaptic connections

**Figure 1:**
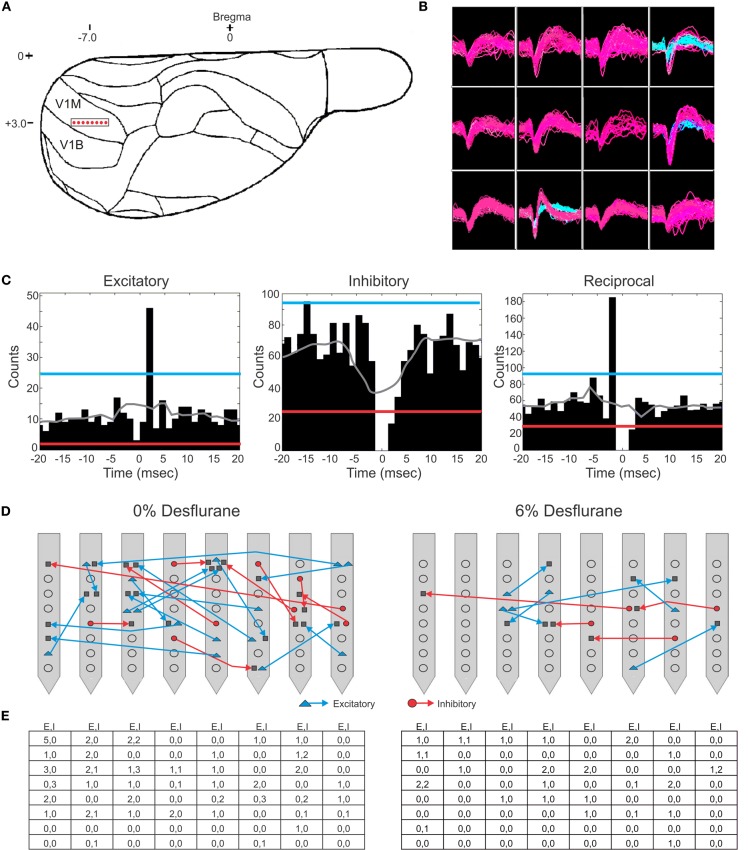
**Schematic of electrode placement and examples of recorded units and connection types. (A)** Electrode placement of the 64-contact neural probe in the rat primary visual cortex monocular region (V1M) in the right hemisphere. Each dot represents the approximate location of an electrode shank. Schematic is overlaid on a stereotaxic drawing obtained from the Paxinos rat brain atlas. **(B)** Example of recorded spike waveforms from 12 channels in one experiment. Color waveforms represent online sorting of units during acquisition. **(C)** Spike cross-correlograms for excitatory, inhibitory and reciprocal connections. Thresholds are represented for excitatory connections (blue line), inhibitory connections (red line), and jittered mean displayed as gray trace. Bin size is 1.3 ms. The gap in the center bin reflects the blanking period of spike sampling for connections observed within the same electrode contact. **(D)** Illustration of excitatory (blue) and inhibitory (red) connections superimposed on a map of electrode contacts during wakefulness (0% desflurane) and unconsciousness (6% desflurane) from all experiments. Presynaptic cell putatively defined as pyramidal cell (blue triangle), interneurons (red circle), or unclassified (gray square). In some cases multiple units are shown at the same contact. For greater clarity, connections between electrode contacts only are shown. **(E)** Number of classified within-contact excitatory and inhibitory (E,I) connections during wakefulness (left) and unconsciousness (right).

Spontaneous extracellular spikes were recorded using 64-contact multishank neural probes chronically implanted in the primary visual cortex (Figure 1A). Each electrode shank spanned the entire depth of the cortex, recording from eight equally spaced depths and eight equally spaced positions. Spikes were detected at approximately half of the electrode contacts (54 ± 16%). Spike sorting yielded one to three units from each electrode contact (Figure 1B). In seven rats during wakefulness, 434 active units with spike rates of at least 1 s^−1^ were recorded. The number and spike rate of units decreased with the anesthetic concentration (*p* < 0.05, linear trend, Table 1).

**Table 1:** **Properties of classified units and connections used for CCG analysis from seven experiments**.

	**Desflurane concentration**			
	**0%**	**2%**	**4%**	**6%**
**ALL**				
Units^*^	434	309	346	271
Spike rate, 1/s^*^	5.5 (4.9, 6.5)	4.2 (3.6, 4.8)	3.3 (2.9, 3.7)	2.8 (2.4, 3.2)
**CLASSIFIED**				
Connections, all^*^	94	67	53	44
Excitatory	60 (64%)	39 (58%)	28 (53%)	31 (71%)
Inhibitory	34 (36%)	28 (42%)	25 (47%)	13 (30%)
Putative cells, all	90	47	50	42
Pyramidal cell	58 (64%)	28 (60%)	27 (54%)	31 (74%)
Interneuron	32 (36%)	19 (40%)	23 (46%)	11 (26%)
Pyramidal spike rate, 1/s	3.8 (3.3, 5.5)^**^	3.5 (2.5, 6.2)	3.5 (2.2, 3.9)	2.6 (1.7, 3.4)
Interneuron spike rate, 1/s	6.3 (4.9, 7.9)	3.4 (1.6, 5.8)	4.0 (2.5, 5.2)	3.6 (1.4, 5.2)

Number in parentheses indicates percent of all connections and cells. Spike rates are median with 95% confidence intervals. A significant linear decrease in the number of units and unclassified spike rates with desflurane was present. A significant difference in the spike rates of putative pyramidal cells and interneurons was observed at wakefulness.

* p < 0.05, linear trend;

** p < 0.05, Mann–Whitney.

Putative excitatory and inhibitory monosynaptic connections were identified by CCG analysis from the counts of correlated spiking between each possible pair of units at various time lags. Examples of CCG corresponding to excitatory, inhibitory, and reciprocal functional connections are illustrated in Figure 1C. The mappings of classified monosynaptic connections found between and within electrode contacts in wakefulness and at the deepest anesthesia level are illustrated in Figures 1D and 1E. In wakefulness, a total of 94 connections were found. This number represents approximately 0.5% of all possible unit pairs. The majority of connections were excitatory (ratio: 1.82 ± 0.71). Anesthesia reduced the number of all connections (*p* < 0.05, linear trend, Table 1).

The CCG analysis also classifies the presynaptic unit as a putative pyramidal cell or interneuron depending on whether it forms an excitatory or inhibitory connection. Putative pyramidal cells fired at a lower rate (median: 3.76, 95% CI: 3.25–5.48) than interneurons (median: 6.27, 95% CI: 4.87–7.91) during wakefulness, and their spike rate distributions were significantly different (*p* < 0.01, K–S, data not shown). In addition, a significant difference (*p* < 0.05, M–W) between the spike rates of putative pyramidal cells and interneurons was present after one outlier was removed (>3 SD). The number of both cell types was reduced with deepening anesthesia (Table 1).

### Spatial distribution of monosynaptic connections

**Figure 2:**
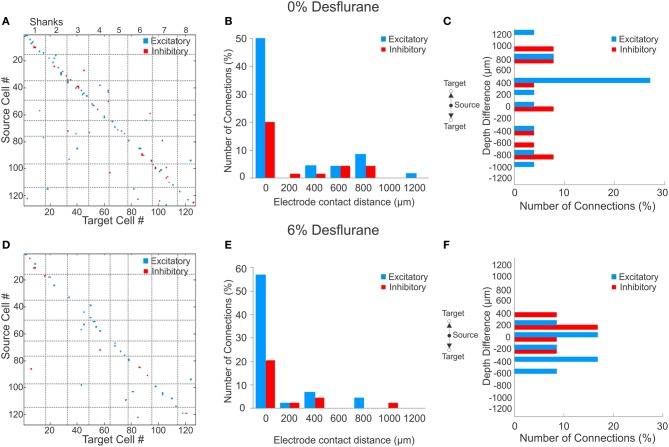
**Excitatory and inhibitory connections at wakefulness and under anesthesia. (A)** Matrix of observed excitatory and inhibitory connections for all rats combined at wakefulness arranged by order of mapping. Cells were numbered from 1 to 127 based on their position of their electrode contact within the array, and arbitrarily within electrode contacts, with the result that consecutive numbers are mapped to neighboring cells. Points on the diagonal represent within-electrode connections, points near the diagonal represent within-shank connections, and points far off the diagonal represent between-shank connections. Most excitatory (blue) and inhibitory (red) connections are found along the diagonal representing connections within or near the same contact. **(B)** Distribution of distance between source and target units during wakefulness. The number of connections in each bin is normalized to the total number of observed connections (excitatory + inhibitory). Most excitatory and inhibitory connections are found within the same electrode contact (73 and 64%, respectively). **(C)** Distribution of depth (source depth—target depth) of excitatory and inhibitory connections from different electrode contacts at wakefulness, corrected for angle of insertion. Excitatory connections project to superficial layers. **(D)** Connection matrix during unconsciousness. **(E)** Distance distribution of functionally connected neuron pairs at unconsciousness normalized to total number of connections. Similar to the wakeful condition, both excitatory and inhibitory connections are mainly found within the same electrode contact (81 and 69%, respectively). **(F)** During unconsciousness the connection depth was limited to shorter distances.

During wakefulness, most connections were short-range, within 200 um (Figure 2A), and most inhibitory and excitatory connections were confined to the same electrode contact at 73 and 64%, respectively (Figure 2B). This was similar at the deepest anesthetic level (6% desflurane, Figure 2E), where short-range excitatory and inhibitory connections were present at 81 and 69%, respectively. However, the number of long-range connections was noticeably smaller than in wakefulness (Figure 2D). During wakefulness, most excitatory connections projected from deeper to more superficial layers, whereas inhibitory connections were widespread, spanning nearly all cortical layers (Figure 2C). During anesthesia, the connections were limited to a shorter intralaminar span (Figure 2F).

To investigate if a reduction in active units contributed to the paucity of long-range connections, we compared the statistical distribution of the Euclidean distance of all possible connections among the measured units in wakefulness and anesthesia (data not shown). We found that the distributions were essentially identical (*p* = 0.74, K–S) implying that the reduction in connection distances was not due to a reduction in the number of active units.

### Connection probability and connection strength

**Figure 3:**
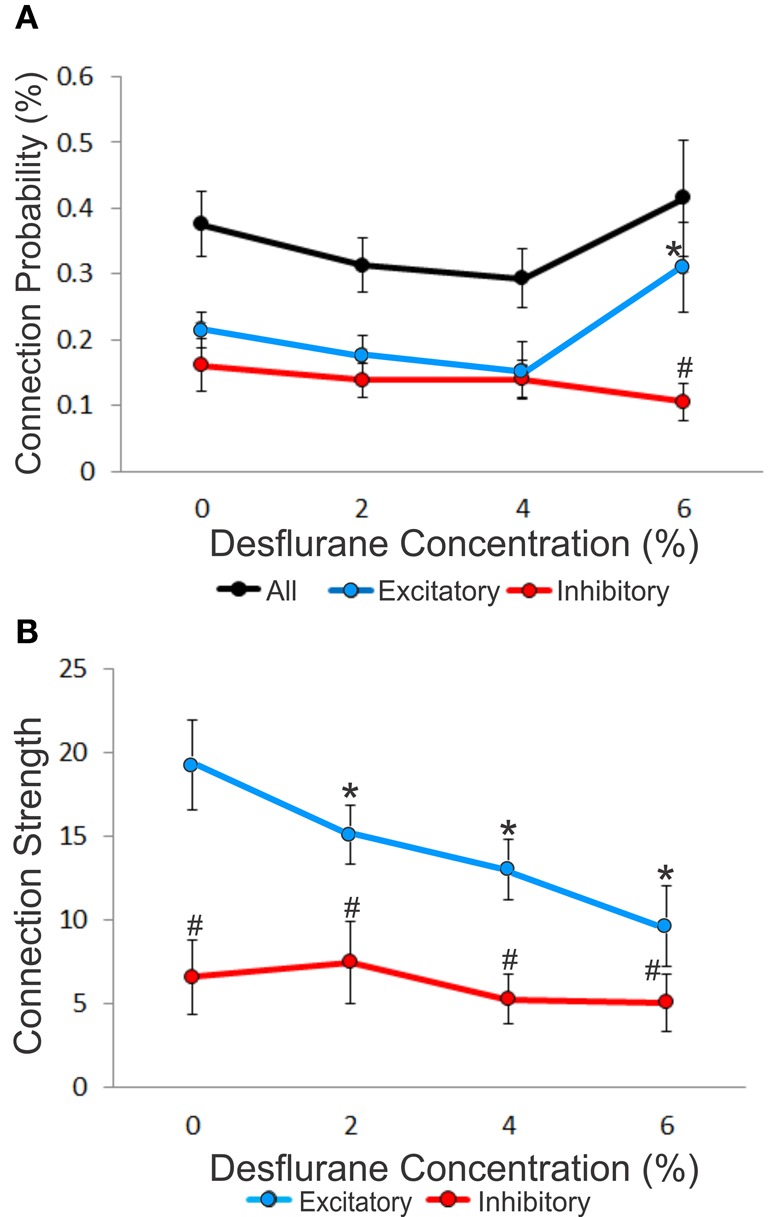
**Concentration-dependent effect of desflurane on connection probability and connection strength. (A)** Effect of desflurane concentration on the percent connected over all possible connections. A significant difference on excitatory and inhibitory connections at 6% desflurane is observed, 3:1 (#*p* < 0.01, Tukey–Kramer). At 6% desflurane, significant increase in excitatory connection probability is observed from the 4% concentration level (^*^p < 0.05, Bonferroni). Data are represented as mean ± SEM. **(B)** Desflurane effect on connection strength. A significant linear decrease in excitatory connections strength (^*^p < 0.05, linear trend) with desflurane is present. A significant difference between the strength of excitatory and inhibitory connections is present at all desflurane concentrations (#*p* < 0.001, RM-ANOVA). Data are represented and mean ± SD.

An unbiased measure of functional connectivity is connection probability, defined as the number of observed monosynaptic connections relative to the number of all possible pairs of the recorded units (Figure 3A). Anesthesia exerted a differential effect on excitatory and inhibitory connection probabilities, as indicated by a significant interaction term (*p* < 0.05, RM-ANOVA). This effect was due to a significantly higher probability of excitatory vs. inhibitory connections (ratio: 2.95, *p* < 0.01, T–K) at the 6% desflurane level. The higher excitatory to inhibitory connection probability at 6% resulted from a significant increase in the excitatory connection probability from the 4% concentration level (*p* < 0.05, Bonferroni). There was no difference in connection probability between wakefulness and the two lighter levels of anesthesia.

We also examined connection strength, measured by the normalized height of the CCG peaks (Figure 3B). This quantity characterizes the efficacy of monosynaptic spike transmission. Anesthesia reduced excitatory connection strength in a dose-dependent manner (*p* < 0.05, linear trend). There was no change in the strength of inhibitory connections.

**Figure 4:**
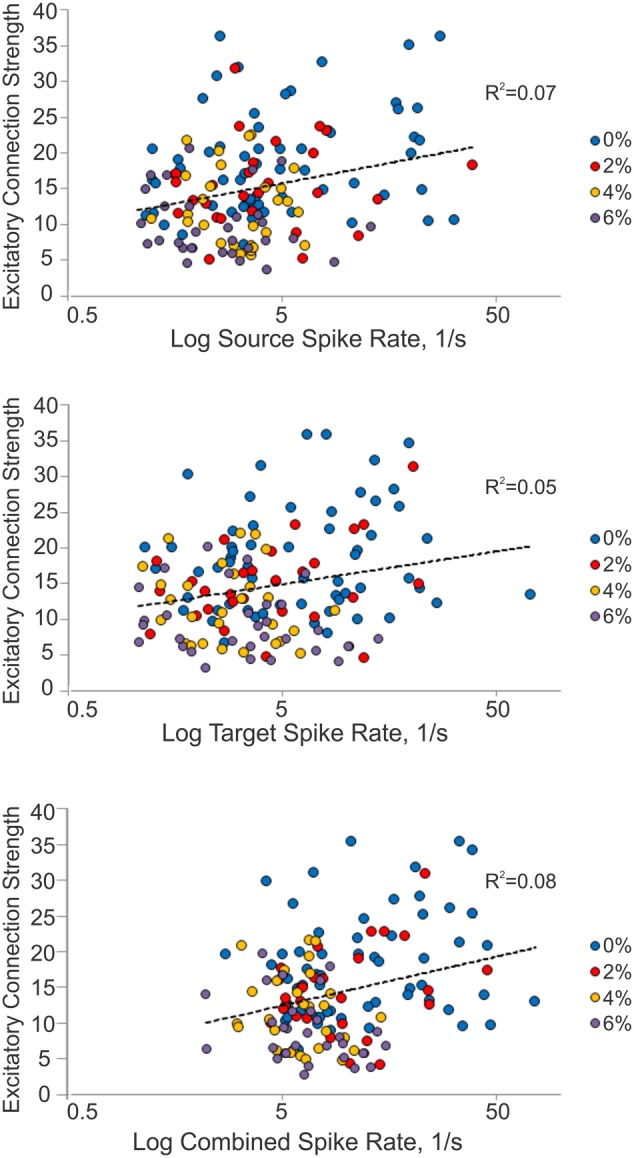
**Relationship between connection strength and spike rate**. Scatterplot of excitatory connection strength and source (top), target (middle) and combined (bottom) spike rates from all experiments. Source spike rate (*R*^2^ = 0.07), target spike rate (*R*^2^ = 0.05), or combined spike rate (*R*^2^ = 0.08) is not an indicator of connection strength.

We considered the possibility that excitatory connection strength might decrease because of the reduced spike rate, reducing the height of the correlation peaks in the CCG. We examined this by constructing a correlation plot of the connection strength and the corresponding spike rate of each connected unit or unit pair (Figure 4). The results showed a very low correlation between connection strength and spike rate (source: *R*^2^ = 0.07, target: *R*^2^ = 0.05, combined: *R*^2^ = 0.08).

To examine this question further, we sought to determine if a decrease in spike rate of either the presynaptic (source) or the postsynaptic (target) unit alone or both could alter the detectability of excitatory connections. To this end, we chose 13 classified excitatory connections of highly spiking source or target units, and decimated the number of spikes in the source, target or both units by 0, 50, 80, 90, or 95%. The CCG analysis was then repeated on all decimated data. The results showed that even at relatively low spike rates (<4 spikes/s), the range of connection strength remained large (5–35, standardized peak height) suggesting an independence of the connection strength from spike rate (data not shown).

## Discussion

This study applied CCG analysis for the first time to investigate the concentration-dependent effects of desflurane anesthesia on putatively classified monosynaptic excitatory and inhibitory functional connections *in vivo*. We found that anesthesia decreased the number of active units and the absolute number of functional connections they formed. Anesthesia also reduced excitatory connection strength that reflects the efficacy of synaptic transmission. Nevertheless, at a depth of anesthesia that purportedly corresponds to unconsciousness, a significant increase in the ratio of excitatory and inhibitory connection probabilities occurred. The latter change suggests an imbalance of excitatory and inhibitory functional connectivity that may indicate abnormal synaptic communication patterns in the state of suppressed consciousness.

Desflurane was chosen for this study because it is a modern and commonly used anesthetic with favorable pharmacokinetic and pharmacodynamic properties and minimal cardiovascular side effects (Eger and Johnson, 1987). The anesthetic actions of desflurane are in most respects similar to those of isoflurane (Rehberg et al., 1999; Murrell et al., 2008), with the exception of its rapid equilibration, which makes desflurane a preferred choice for experiments to be performed at multiple steady-state anesthetic depths in the same experimental setting.

The effect of anesthesia on spike transmission probabilities in intact cortical neuronal networks, *in vivo*, has not been investigated. Previous studies using *in vitro*, whole cell or single-channel recordings have established that most anesthetics enhance inhibitory and suppress excitatory synaptic transmission by modulating ligand-gated ion channels (Pearce et al., 1989; Ries and Puil, 1999; Pittson et al., 2004). Our results are consistent with these observations in that inhibitory connection strength was resistant to desflurane, whereas excitatory connection strength was decreased in a concentration-dependent manner. Minor differences with the *in vitro* data, such as the lack of enhancement of inhibitory connection strength, are understandable due to the recurrent nature of excitatory and inhibitory interactions in local circuits *in vivo*.

In contrast, the significantly higher excitatory to inhibitory connection probability (E/I balance) at 6% desflurane anesthesia was unexpected. This effect could be due to a change in spike patterns or circuit properties, perhaps to a reduced absolute number (but not strength or efficiency) of inhibitory connections. The difference between the changes in excitatory connection probability and excitatory connection strength is understandable given the different nature of the two parameters. Connection probability measures the frequency of occurrence of functional connections relative to the number of all possible pairs of active units. Connection strength, on the other hand, characterizes the efficacy of spike transmission for each identified functional connection. Thus, it is possible to encounter higher connection probability at lower transmission efficiency.

We also found that the decrease in connection strength could not be directly accounted for by the decrease in spike rate of either the source or the target cells. One explanation for the decreased connection strength may be the change in firing pattern of the presynaptic cell. Short interspike-intervals between pairs of spikes in the presynaptic cells have been shown to more robustly discharge their postsynaptic target (Usrey et al., 1998; Kara and Reid, 2003). Anesthetic modulation of spike pattern variability and its consequent effect on connection strength and connection probability may be investigated in the future.

The change in E/I balance observed at 6% desflurane concentration is important. It has been suggested (Shew et al., 2011) that information transmission and information capacity are maximized at intermediate E/I. An alteration of the E/I balance, in particular elevated E/I, may impair information processing as observed in psychiatric disorders (Yizhar et al., 2011), suppress memory retrieval and recall (Wang and Zochowski, 2012) and reduce sensory-motor integration as observed in evoked responses with lower doses of anesthetics (Populin, 2005). An elevation of E/I from its optimal value may represent insufficient suppression of excitation by inhibition, and was shown to result in excessive correlation, also referred to as hypersynchrony, between neurons (Shew et al., 2011). In fact, our results revealed an increase in excitatory connection probability at the deepest anesthetic level. As suggested by Buzsaki and colleagues, the lack of inhibition could create an unstable system resulting in an avalanche of excitation (Buzsaki, 2006; Buzsaki et al., 2007) that is incompatible with meaningful information processing. Stereotypic hypersynchronous activity is commonly observed in deep anesthesia characterized by electroencephalography (EEG) burst-suppression and is thought to have limited information capacity (Alkire et al., 2008). In burst-suppression, the cortex displays brief periods of increased activity followed by electrically silent periods. It is accompanied by cortical hyperexcitability through reduced inhibition, therefore causing a shift in the E/I balance (Hudetz and Imas, 2007; Amzica, 2009; Ferron et al., 2009). In our experiments at desflurane concentrations up to 6%, there was no burst-suppression in recorded local field potentials, suggesting that the state of hyperexcitability was not attained. It is possible that neuronal firing may have acquired a bursting pattern—as another form of hyperexcitability, although this was previously observed under deep urethane anesthesia only (Erchova et al., 2002). This possiblity should be tested in additional studies in the future.

The present results demonstrate that CCG analysis can extract putative monosynaptic connections of functionally interacting neuron pairs at distances up to 1200 μm in the rat visual cortex in both wakefulness and under anesthesia. We also observed a more pronounced deflection in the jittered CCG histogram of inhibitory than excitatory connections. This is consistent with previous *in vivo* and *in vitro* studies and reflects the slower timecourse of inhibitory postsynaptic potentials (PSPs) relative to excitatory PSPs (Thomson et al., 1996; Tamas et al., 1997; Bartho et al., 2004; Fujisawa et al., 2008). Similar to previous studies (Bartho et al., 2004; Buzsaki, 2004; Fujisawa et al., 2008), most functional connections were close-range (<200 μm) and found within the same electrode contact. During wakefulness, most excitatory connections projected upward toward more superficial layers consistent with that seen in the auditory cortex of identified pyramidal cells (Crochet and Petersen, 2009; Sakata and Harris, 2009). In the anesthetized condition, the spread of excitatory activation was confined to smaller cortical depths suggesting a reduction in information transmission across cortical layers. The reduction in the spatial dispersion of monosynaptic connections may therefore be another indication of reduced cortical communication and integration associated with the anesthetic induced unconsciousness.

We referred to anesthesia at 6% desflurane as a state of unconsciousness. Arguably, consciousness cannot be directly assessed; we can only measure a behavioral surrogate. In rats, the righting reflex is a widely used behavioral index of consciousness because it is abolished at equivalent anesthetic concentrations to those that abolish response to verbal commands in human subjects (Franks, 2008). The desflurane concentration that suppresses the righting reflex has been previously determined as 4.6 ± 0.45% (Imas et al., 2005b). The experiments were conducted starting with the anesthetized condition and finishing with the wakeful condition. We chose this order of conditions to the initial threshold selection for spike detection under anesthesia, when signal-to-noise ratio was optimal. Thus, strictly speaking, we investigated the neuronal events associated with regaining as opposed to losing consciousness. During emergence from anesthesia, the threshold for righting reflex may be slightly lower than during induction (Friedman et al., 2010), indicating hysteresis or “neuronal inertia”. Because our experiments were conducted under steady-state conditions with relatively long equilibration periods before each recording, a hysteresis effect was very unlikely. In preliminary studies with similar equilibration periods, we observed no significant difference in spike rate or interspike intervals between induction and emergence conditions at the same anesthetic concentration. Therefore, 4–6% is a good estimation of range of desflurane concentration at which a reversible transition between consciousness and unconsciousness occurred.

In general, anesthetic drugs target various ligand-gated and voltage-gated ion channels that regulate synaptic transmission (Rudolph and Antkowiak, 2004; Franks, 2006; Alkire et al., 2008) and it is difficult to extrapolate the effect of one agent to that of another. Depression of neuronal excitability has been observed with various anesthetics (Hentschke et al., 2005; Schumacher et al., 2011), thought to be primarily caused by enhanced synaptic inhibition at γ-aminobutyric acid A (GABA_A_) receptors (Banks and Pearce, 1999; Bieda and Maciver, 2004) producing hyperpolarization. A suppression of inhibitory neurotransmitter release (Maclver et al., 1996) and the anesthetic modulation of sodium and potassium channels (Hemmings et al., 2005) may also contribute to reduced excitability. Previously, we showed a suppression of baseline and long-latency cortical neuronal responses to stimuli under desflurane *in vivo* (Hudetz et al., 2009). Inhalational anesthetics such as isoflurane and sevoflurane, as well as intravenous anesthetics propofol and midazolam, and perhaps α-chloralose (Garrett and Gan, 1998), primarily GABA_A_ potentiators similar to desflurane, may produce comparable results. Generalization to other types of anesthetics, such as ketamine or urethane, with substantially different ionic mechanisms and targets (Harrison and Simmonds, 1985; Hara and Harris, 2002; Sceniak and Maciver, 2006) is not straightforward.

As with all similar studies, a recognized technical limitation is the undersampling of the neuronal population. Although we were able to simultaneously record approximately 70 units during wakefulness in each experiment, this number represents a small percentage of active neurons in the sampled region. Because recorded spike amplitudes are attenuated exponentially with distance, 60–100 neurons could be reliably recorded within a 60 μm radius in the rat hippocampus and medial prefrontal cortex (Buzsaki, 2004; Fujisawa et al., 2008). Assuming similar spike amplitude attenuation in the visual cortex, under optimal conditions, we were able to isolate 1–3 units per electrode contact, representing 1–3% of the total possible units. Possible reasons for the relatively low number of recorded cells in our experiments include the potential damage to cells by insertion of the electrode and the possible insulated nature of silicon probe shank that reduces the number of observable neurons (Moffitt and McIntyre, 2005). Furthermore, the spike rate decreased in a concentration-dependent manner, thus reducing the number of active units (>1 s^−1^) used for CCG analysis. The limited number of recorded cells and concentration-dependent change in spike rate may also account for the relatively low percentage of classified monosynaptic connections relative to all possible connected cell pairs as identified by CCG analysis, consistent with previous findings (Csicsvari et al., 1998; Fujisawa et al., 2008). Another limitation of the pairwise CCG analysis is that it cannot account for the effects of possible indirect connections (Gerstein and Perkel, 1969) although the time scale of interactions (~1–5 ms) makes the contribution of multi-synaptic effects unlikely. Recent studies have shown that pairwise analysis may represent the correlated states of a network surprisingly well both *in vitro* (Schneidman et al., 2006; Shlens et al., 2006; Tang et al., 2008) and *in vivo* (Yu et al., 2008). Therefore, the pairwise CCG method should represent a reasonable first approximation of population activity.

In summary, our results demonstrate that general anesthesia by desflurane at a concentration that induces unconsciousness alters the excitatory/inhibitory balance of monosynaptic interactions in rat visual cortex neurons *in vivo*. The elevation of the excitatory-inhibitory balance may result from altered spike firing variability, therefore reducing the efficacy of excitatory transmission among the neurons. Overall, elucidating the effect of general anesthesia on functional communication between cortical neuronal cells should help better understand how changes in spikes modulate population activity as a function of cortical state and awareness.

## Materials and methods

The proposed experimental procedures and protocols were reviewed and approved by the Institutional Animal Care and Use Committee. All procedures conform to the *Guiding Principles in the Care and Use of Animals* of the American Physiologic Society and are in accordance with the *Guide for the Care and Use of Laboratory Animals* (National Academy Press, Washington, DC, 1996). All efforts were made to minimize the number of animals used and their suffering.

### Electrode implantation

Seven adult male Sprague-Dawley rats were kept on a reversed light–dark cycle in dedicated rooms of the Animal Resource Center for at least one week prior to physiological experiments. On the day of the aseptic surgery, the rat (260–440 gm) was anesthetized using isoflurane (Abbott Laboratories, Chicago, IL) in an anesthesia box. The animal's head was then secured in a rat stereotaxic apparatus (Model 900, Kopf Instruments, Tujunga, CA) and a gas anesthesia adaptor (Stoelting Co., Wood Dale, IL) was placed over the snout to continue anesthesia at ~2.0% isoflurane. Body temperature was rectally monitored and maintained at 37°C via an electric heating pad (TC-1000, CWE Inc., Ardmore, PA). The antibiotic, Enrofloxacin (10 mg/kg s.c.), was administered prior to surgery onset. The dorsal surface of the head was prepared for sterile surgery with betadine and alcohol. Bupivicaine, a local anesthetic, was injected under the skin prior to surgery. A midline incision was then made and the skin was laterally reflected. The exposed cranium was gently scraped of connective tissue and any bleeding was cauterized.

A multishank, 64-contact microelectrode array (5 mm length, 200 μm electrode spacing, 200 μm shank spacing, Neuronexus Technologies, Ann Harbor, MI) was chronically implanted stereotaxically within the monocular region of the visual cortex, V1M (7.0 mm posterior, 3–3.5 mm lateral, relative to bregma) as illustrated in Figure 1A. To implant the microelectrode array, a craniotomy of rectangular shape of approximately 2 × 4 mm was prepared using a low speed, compressed air-driven dental drill and bur No. FG 1 (Sullivan/Schein Dental, Melville, NY). The exposed dura mater was resected and the electrode array was inserted using a micromanipulator. The array was subsequently advanced at increments of 10 μm to a depth of approximately 2.1 mm below the brain surface. To secure the neural probe, the perimeter surrounding the electrode probe was covered with silicone gel (Kwik-Sil, World Precision Instruments, Sarasota, FL). A reference wire attached to the neural probe was wrapped around a cranial steel screw located between bregma and lambda (~4.0 mm posterior, ~2.0 mm lateral, relative to bregma) in the opposite hemisphere.

In addition to the implanted electrode, sterilized stainless steel screws (MX-080-2, #0−80 × 1/8″, Components Supply Co Inc, Fort Meade, FL) were placed in the cranium as anchors. The whole assembly was embedded into a nontoxic skull fixture adhesive, Cerebond (MyNeurolab, Saint Louis, MO), with only the IC connectors protruding from the skull fixture adhesive cap. Analgesic (5 mg/kg carprofren s.c.) was administered postsurgery. The animal was then returned to the housing cage in the animal facility. Carprofren (5 mg/kg s.c. once daily) was administered for 2 days and enrofloxacin (10 mg/kg s.c. once daily) for 7 days. The animal was then observed for 7–10 days for any infection or other complications.

### Experimental protocol

Following recovery, the rat was placed in a cylinder anesthesia chamber. The chamber was then sealed and ventilated with a heated, humidified gas mixture of 30% O_2_, balance N_2_. The room was then darkened and the rat was allowed to freely move around in the box for about 1 h to accommodate to the environment. After the accommodation period, the electrode array was then connected to a headstage with its wire bundles connected to a preamplifier (Blackrock Microsystems, Salt Lake City, UT) outside the anesthesia box.

Spontaneous UA was recorded using a 128-channel neural acquisition system (Blackrock Microsystems, Salt Lake City, UT). Extracellular neural activity was auto-thresholded using a root mean square multiplier of −6.25 and kept constant throughout the experiment. Spiking activity was analog filtered from 250 to 7500 Hz and digitally sampled at 30 kHz.

Recording was performed first under anesthetized conditions and then in wakefulness. Three anesthetic concentrations were used at which rats were either unconscious (6%), moderately anesthetized (4%), or lightly sedated (2%). Continuous monitoring of the anesthetic concentration was performed using a POET II monitor (Criticare Systems, Waukesha, WI). Since monitoring accuracy is 0.1%, an indication of the target or target ±0.1% concentration was accepted. An equilibration time of 15–20 min after a decrease in concentration was allowed before recording of spontaneous activity. In each condition, spontaneous UA was recorded for 10 min.

### Spike train analysis

Movement artifacts were identified as synchronous time segments across all channels and manually removed. On average, 4.9 ± 1.3% of the data contained signal artifacts due to chewing, twitching, or grooming. An 8–10 min segment of artifact-free spontaneous extracellular UA was extracted from the recordings at each state for postprocessing and further analysis.

At each concentration, PowerNAP (OSTG, Inc., Fremont, CA), an open-source software, was used to sort the spike waveforms at each contact into individual neuronal units. This offline spike sorter software applies principal component analysis (PCA) along with various clustering methods for sorting. PCA determines the linearly dependent factors in the spike waveform data and transforms them into an ordered set of orthogonal basis vectors that capture the direction of the largest variation (Fee et al., 1996; Hudetz et al., 2009). A scatterplot using the first two principal components was then constructed, and K-means clustering analysis was used to define the cluster boundaries of individual units. Occasional remaining outliers were removed manually, if necessary.

### Cross-correlogram analysis

CCG analysis is a linear statistical assessment of the interdependencies between pairs and represents how two signals relate with one another as a function of time displacement. It has been applied to indirectly classify monosynaptic connections as either excitatory or inhibitory based on the functional interaction dynamics between neuronal cell pairs (Csicsvari et al., 1998; Bartho et al., 2004; Fujisawa et al., 2008). CCG is calculated as the time difference of spike occurrences (cross-interspike interval) between a reference spike and the target spike train. Here we used a time window interval of [−20, +20] ms with a 1.3 ms bin size to produce a count histogram of the calculated cross-interspike intervals.

In order to eliminate short-time scale chance correlations while retaining larger-time scale (i.e., spike rate) information, a jitter resampling method was performed (Fujisawa et al., 2008; Quilichini et al., 2010). A simulated randomized spike train was produced by independently and randomly “jittering” or shifting the occurrence time of each spike in the target spike train within a small uniformly distributed time interval of −5 to +5 ms. CCG analysis was then performed on the reference spike train and the jittered spike train. The jittering method was performed 1000 times, yielding 1000 surrogate data sets. The variation produced by the jittered CCG data sets provided the confidence intervals for the number of counts in each bin. Global thresholds of 97% confidence interval were determined from the maximum and minimum of each jittered surrogate CCG and used for classification of significant monosynaptic connections. Monosynaptic connections were identified by the presence of a significant CCG peak height or trough observed within the delay interval of [+1.3, +5.2] ms. Significance was determined with respect to the global thresholds (Csicsvari et al., 1998; Bartho et al., 2004; Fujisawa et al., 2008). The count values in original CCG histogram that surpassed or fell below twice the global threshold within this short latency interval indicated a direct excitatory or inhibitory monosynaptic connection, respectively. For the units that were recorded from the same electrode contact, the time zero bin was excluded from the analysis due to the built in blanking period of the spike detection system. CCG was calculated using the sigTool toolbox (Lidierth, 2009) in MATLAB R2007b (Mathworks, Natick, MA).

The effect of desflurane anesthesia on excitatory and inhibitory connections was determined as the total number of putatively identified monosynaptic connections normalized to the total number of possible connections at each anesthetic concentration. The Euclidean distance of the connected neuronal pairs was determined based on their electrode locations. A histogram of the distance lengths for each connection type was then created and normalized to the total count of excitatory and inhibitory connections. The vertical distance from the source to the target cell, representing the connection depth, was also calculated for excitatory and inhibitory connections at each concentration and a histogram was produced.

Connection strength represents the efficacy of spike transmission between each pair of cells and was defined as the standardized peak height in the CCG. Specifically, the absolute difference between the number of spikes in the peak or trough of the CCG histogram and the jittered mean was taken and divided by the jittered standard deviation (Fujisawa et al., 2008). Based on the type of connections revealed by the CCG analysis, each presynaptic cell was indirectly classified as a putative pyramidal cell or interneuron. The majority of pyramidal cells fire at lower frequencies than do interneurons (Csicsvari et al., 1998). The distribution of spike rates of the two putatively classified cell types was compared.

### Statistical assessment

The effects of desflurane on baseline firing rates and number of active units, excitatory and inhibitory connections were estimated using RM-ANOVA test with the anesthetic concentration as a fixed factor and the subject (rat) as a random factor. Deviation from the zero slope was tested using a linear trend planned comparison test. The spike rate distribution of classified pyramidal cells and interneurons at waking across all rats were compared using a Kolmogorov–Smirnov (K–S) test. A significant difference in the spike rates of putative pyramidal cells and interneurons was tested using a Mann–Whitney (M–W) test. The concentration-dependent effects of desflurane on the percentage and strength of connections was tested with RM-ANOVA with type (excitatory or inhibitory) and desflurane concentration as within-factors, the subject (rat) as random variable, and the percentage of connections or connection strength as the response variable. When the interaction term was significant, the component effects were further examined using Tukey–Kramer Multiple-Comparison test (T–K) or a Bonferroni test. Statistical analyses were performed using NCSS 2007 (NCSS, Kaysville UT). Data are presented as ± standard deviation (SD) from the mean or median with 95% confidence intervals.

### Conflict of interest statement

The authors declare that the research was conducted in the absence of any commercial or financial relationships that could be construed as a potential conflict of interest.
